# 

**Published:** 1840-10

**Authors:** 


### CONTENTS

OF NO. X.

---

### ORIGINAL COMMUNICATIONS.

ESSAYS AND CASES.

ART. I.—An Essay on Tenotomy; with illustrative Cases. By S. B. RICHARDSON, M. D., Lecturer on Anatomy and Surgery, &c. Louisville, Ky. - - - - - - - 245

ART. II.—A Fourth Supplementary Catalogue of the Plants of Kentucky. By C. W. SHORT, M. D., Professor of Materia Medica and Medical Botany in the Medical Institute of Louisville. - - - - - - - - 283

REVIEWS.

ART. III.—Elements of Pathological Anatomy; illustrated by numerous engravings. By SAMUEL D. GROSS, M. D. Boston: 1839. - - - - - - - - - 289

SELECTIONS FROM AMERICAN AND FOREIGN JOURNALS.

Fatal effects from the Acetate of Lead given in large amount for Phthisis. By Dr. BICKING, of Mulhouse. - - - - - 305

On the transformation of Calomel into Corrosive Sublimate. By M. Mialhe. - - - - - - - - - - - - 307
Emetics of Ipecacuan. in Hemorrhage. - - - - - - 309
Cure of Strabismus. - - - - - - - - - - 310
Red Sulphur Springs. - - - - - - - - - - 310

ORIGINAL INTELLIGENCE.

Lancaster Medical Institute. - - - - - - - 315
Dover's Powder modified. - - - - - - - - - 316
Temporary Hemiplegia. - - - - - - - - - 317
Progress of the Temperance Reform. - - - - - - 318
Louisville Marine Hospital. - - - - - - - - 319
Tea and coffee as the cause of Sick-Headache. - - - - 320
Velpeau's Operative Surgery. - - - - - - - - 321
Health of Louisville. - - - - - - - - - - 321
Death of Dr. Perrine. - - - - - - - - - - 321
Tenotomy—a correction. - - - - - - - - - 323

